# Sex and Population Drive Interindividual Variations in a Cognitive Task Across Three Populations of Wild Zebrafish

**DOI:** 10.3389/fpsyg.2022.786486

**Published:** 2022-03-04

**Authors:** Danita K. Daniel, Anuradha Bhat

**Affiliations:** Department of Biological Sciences, Indian Institute of Science Education and Research – Kolkata, Mohanpur, India

**Keywords:** repeatability, interindividual variation, wild zebrafish, population difference, geographic variation, animal behavior, spatial cognition, learning and personality

## Abstract

Animal personality refers to the consistency of variation in behavior among individuals which may be the driving force behind variations in complex behaviors as well. Individual personality could predict how well an organism would perform in behavior and cognition related tasks, as well as survive and thrive in its environment. Therefore, we would expect inter-individual variations in many behaviors, which would persist even if habituation to the experimental setup occurs, which generally results in convergence of behavior (i.e., the difference between individuals becomes less pronounced). Our study used wild-caught zebrafish (*Danio rerio*) from three natural habitats with differing ecological regimes, to understand how consistency and repeatability in specific traits such as boldness, exploration, and spatial ability varies across and within populations even when habituation causes change in behavior. We found that the extent of individual variation differs between populations, with dynamic habitats showing similar repeatability. This indicates that habitat conditions are important drivers of individual variation in addition to other factors, such as sex or size of individuals within populations. Although we found that sex and size played an important role within some populations for some behaviors, in others, the variation was likely caused by other factors (for example, ecological factors such as vegetation and/or resource availability), for which we have not accounted. This study underlines the importance of studying inter-individual differences as the phenomenon that underpins multiple behavioral traits and explains the possible role of environmental and inherent factors that drive these differences.

## Introduction

In order to thrive in its habitat, it is essential for an organism to develop mechanisms that allow it to adapt to local environmental conditions. These adaptations can be behavioral, physiological, morphological or even genetic in nature, resulting in differences among individuals of the same species living in different habitats. Varying selection pressures in these habitats may cause individuals within each population to behave differently, resulting in inter-population variations within the same species (Snell et al., 1988; Bobyn and Brooks, 1994; Uren Webster et al., 2018). Studies on a neotropical rainforest bird (*Pyriglena leucoptera*), showed that birds from populations found in highly fragmented habitats were better able to disperse than birds from intact habitats (Cornelius et al., 2017). Studies on snails, garter snakes and mayflies have shown that populations vary in the way they respond to the same predator (Arnold, 1992; McIntosh and Townsend, 1994; Levri et al., 2012).

Many freshwater fish species form distinct isolated populations that might vary in behavioral traits and this allows for a comparative study of multiple traits between populations. Sticklebacks vary in personality and response to predators, as well as in brain size across populations (Giles and Huntingford, 1984; Bell, 2005; Alvarez and Bell, 2007; Gonda et al., 2011). Different species of perches have also been found to differ across populations, in life history strategies, morphology and even some behaviors, such as catchability and activity (Heibo and Vøllestad, 2002; Svanbäck and Eklöv, 2006; Härkönen et al., 2016; Nakayama et al., 2016).

While most investigations in animal behavior and behavioral ecology have typically focused on deciphering population variations, performing studies on individual variations within and across populations has also become of vital importance. While geographic or inter-population variations in behavior are likely to be adaptations to varying habitat conditions, intra-population variation may be the result of differing strategies employed by individuals within populations, to enhance their survival (Snell et al., 1988; Fraser et al., 2001).

Even within a population, some behaviors are more rigid than others, and are less likely to change for an individual over time and context which causes greater differences between individuals (Bell, 2007; Réale et al., 2007). One potent way to quantify these differences, is in terms of “repeatability” which measures the proportion of behavioral variation that is due to differences between individuals in the population (Boake, 1989; Rudeck et al., 2020). Repeatability has also been studied for a variety of behaviors across multiple taxa, and has been found to vary in many ways depending on a vast range of biotic and abiotic factors (Bell et al., 2009) that are themselves dynamic in nature. A comparison of studies on different fish species shows that repeatability depends on the environmental context. Since ecological conditions can vary across habitats, different populations can also vary in the extent of behavioral repeatability (Killen et al., 2016).

While inter-individual differences have been quantified and characterized for a wide variety of behaviors (Bell et al., 2009), there are few studies investigating repeatability in cognitive tasks within and among populations. However, the links between personality and cognition have been explored in multiple species, which allows for extrapolation of the effect of inter-individual variations on cognitive performance (Revelle et al., 2010; Carere and Locurto, 2011; Boissy and Erhard, 2014; Dougherty and Guillette, 2018). In a mormyrid fish, bolder fish learnt a spatial task faster than shy fish in multiple conditions (Kareklas et al., 2017). Similarly, in guppies, more sociable individuals performed poorly in a shoal size discrimination task (Lucon-Xiccato and Dadda, 2017). However, other studies have indicated that there is no effect of personality on cognitive performance in female guppies tested in a foraging learning task (Kniel et al., 2020). Even if there is no direct effect of personality on cognitive ability, similar factors (such as size and sex) influence both kinds of behavior, which might result in correlated patterns (Barber et al., 2017; Jacquin et al., 2020), indicating that even if personality does not affect cognitive performance, the two behaviors are likely to covary.

The zebrafish (*Danio rerio*) (Hamilton, 1822) is a small, tropical freshwater fish native to the streams of the Indian subcontinent. Since its discovery, it has become a popular model organism in various fields of biology (Engeszer et al., 2007; Spence et al., 2008; Parichy, 2015), and more recently, behavioral variations as well as repeatability in behavior have also been explored among wild zebrafish populations. For instance, wild zebrafish populations have been shown to differ in behavioral plasticity when subjected to environmental manipulation (Bhat et al., 2015) and variation in boldness, exploration, aggression, sociability, and learning abilities have been demonstrated in several wild-caught populations (Roy and Bhat, 2016; Roy and Bhat, 2018a,b).

Since zebrafish are a popular model organism in behavioral neuroscience, there have been multiple studies on their cognitive skills, and several cognitive tests indicative of different kinds of cognitive ability have been extensively characterized and outlined (Meshalkina et al., 2017; Luchiari et al., 2021). In wild zebrafish, spatial cognition has been seen to be shaped by rearing environment and its complexity (Spence et al., 2011; Roy and Bhat, 2016). This has also been found to result in population variation in cognitive ability when individuals are trained to perform in a spatial task (Roy and Bhat, 2018).

Populations of wild zebrafish also vary in terms of repeatability within populations, and the extent of variation is trait-dependent. Individuals from some populations show greater repeatability in feeding latencies across different contexts (Roy et al., 2017). Repeatability in boldness and aggression depends on flow regime and predator abundance in the native habitat of the populations, with populations with higher levels of predation showing greater repeatability (Roy and Bhat, 2018). Studies in lab-bred zebrafish selected for reactive and proactive behavior have shown that reactive fish are more repeatable in exploratory behavior than proactive fish (Baker et al., 2018). Long term studies on repeatability in zebrafish have shown that consistency in inter-individual differences persists for most behaviors. Any drastic changes in behavior are usually caused by extreme stress due to social isolation or other traumatic events (Thomson et al., 2020). However, interindividual variations in aversive learning do not remain consistent across different contexts, and show low repeatability (Mason et al., 2021).

Although previous studies have demonstrated repeatability and its variation across populations in wild zebrafish, persistence of patterns in behaviors related to cognitive performance is still not clear. When organisms are successfully trained in a cognitive task, their performance is likely to change progressively over trials, which might reduce consistency in individual behavior over time. Although repeated testing is required to study consistency over time for all behaviors, most studies do not take habituation or improvement in task performance into account. Our previous study that explored the relationship between personality and cognition in wild-caught zebrafish discovered correlations between the two but did not test for consistency in behavior over repeated trials (Daniel and Bhat, 2020).

Our present study examines intra- and inter-population variations in specific behaviors that pertain to personality and cognitive abilities in fish, and individual traits that likely drive these variations. We address this by training and testing wild zebrafish from three populations which differed in water flow, turbidity, and habitat complexity. Apart from being trained in a spatial learning task, the fish were also observed in an emergence and exploration task. After testing whether overall performance of individual fish in the emergence, exploration and spatial tasks improved over time, our aim was to disentangle whether these behaviors were still repeatable over trials, indicating an intra-individual correlation in performance in the various tasks. This would aid in illuminating patterns in repeatability of behavior, as well as the factors that underpin individual consistency. We aim to examine repeatability in boldness, exploration and performance in a spatial task when repeated testing or training leads to improvement in performance. We also hope to elucidate any difference in repeatability in behavioral traits between populations, as well as any differences in specific factors (such as population, sex, and body size) which contribute to repeatability. Lastly, since the three populations differed from each other in their habitat characteristics (water flow regimes, habitat complexity, turbidity, etc.), sex ratios as well as size variation, we expected to not only find a difference in repeatability, but also differences in the individual factors (i.e., sex and size) that determine behavioral consistency within each population. Specifically, populations with similar habitat dynamics and or/individual trait properties would be expected to exhibit comparable behavioral repeatability.

## Materials and Methods

### Selection of Habitats

Zebrafish populations from three habitats from West Bengal, India, were collected for the study. The habitats were selected considering their environmental factors such as flow regime (speed and volume of water flowing in the stream), dissolved oxygen (amount of dissolved oxygen in water which is available for biological consumption by aquatic species), temperature, influence of human activity, and the temporal variation of the habitat in terms of flow regime and water volume. Total dissolved solids (TDS) was also measured for the habitats, as a proxy measurement for turbidity. The populations have been named and coded based on the name of the closest neighboring town/locality. Properties of each of the habitats are described below:

Cooch Behar (CB)—This habitat has relatively high flow (∼5.3 m/s) and is temporally dynamic as it varies across seasons (monsoon changes size as well as flow parameters of the river), with clear water (TDS ∼ 54–63 ppm) and minimal vegetation, even on the banks. Zebrafish populations are highly abundant and are found in large shoals (∼1,000 individuals), usually closer to the banks where the flow of the river is slower.

Leturakhal (LK)—This is a moderate flow habitat (1.9 m/s), relatively stable with increased water levels during monsoon, but no significant changes to the flow of the water. There is riparian vegetation on the banks and some anthropogenic interference (fishing, bathing etc.), but water is reasonably clear (TDS ∼ 234–256 ppm), except during the monsoon, when rainfall causes a disturbance, or in areas with regular human disturbances. Moderately large zebrafish shoals (∼200–300 individuals) are found close to the banks, usually near outcroppings or vegetation.

Kali Bazar (KB)—Water in this habitat is stagnant, with almost no flow (0.003 m/s). This is also an extremely dynamic habitat since it dries up in the summer and fills up during the monsoon due to runoff from paddy fields. There is no floating vegetation on the water or riparian vegetation on the banks, but a canopy cover is present due to trees growing beside the stream. It is highly polluted by human activities in its vicinity, and water is never clear (TDS ∼ 543–573 ppm). Very small zebrafish shoals (10–15 individuals) are found all over the habitat but are relatively less abundant.

There are also significant distance and topographical barriers between the three habitats which would prevent any geographical overlap of the populations (CB-KB: ∼395 km; KB-LK: 165 km; CB-LK: ∼510 km).

### Collection and Maintenance of Populations

Fish were collected from each habitat in the post-monsoon season (October to February 2018–2019), when zebrafish abundance is known to be at its peak (Spence et al., 2008) using drag nets and transported to the lab in large, aerated plastic bags. They were maintained in the lab at a temperature of 23 ± 2°C and in a light:dark cycle of 12:12 h, in large, bare glass tanks (60 cm × 30 cm × 30 cm; water level–15 cm) containing filtered water at a density of ∼150 (mixed-sex) fish per tank. Loose or reconstituted freeze-dried blood worms were provided as food *ad libitum* once a day.

After their transport to the laboratory, individual populations were maintained in separate stock tanks for a minimum of 2 months for acclimatization and also to ensure that all individuals grew into sexually mature adults.

### Experimental Setup and Protocol

Forty eight hours prior to the start of experiments, individual test fish were transferred into a tank with mesh compartments (15 cm × 12 cm × 15 cm; water level—10 cm) placed within the tank, allowing for exchange of visual and olfactory cues between individuals. Each mesh compartment was numbered, and housed a single fish, with other individuals from the same population in the adjoining compartments. This ensured minimal isolation related stress, while allowing for tracking of individuals over experimental trials. Each population was isolated and tested in two sets, with each set consisting of 20 individuals (10 males, 10 females). All the individuals in a set were tested on the same day. Individuals from LK were tested first, followed by KB and CB, and both sets of each population were tested consecutively without any gap between them.

**FIGURE 1:**
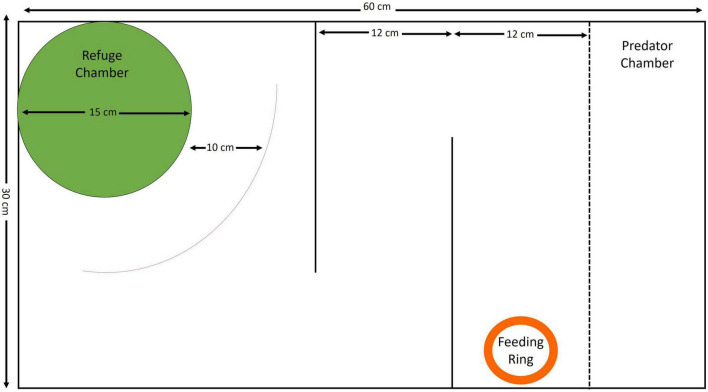
Schematic representation of the setup (top view) with dimensions of each section of the experimental arena.

The experimental setup consisted of a refuge chamber at one end, a simple maze, and a predator chamber at the other end of the tank (Figure 1). The refuge chamber was a cylindrical compartment 15 cm in diameter with a 7 cm × 7 cm window on one side and a roof for shelter. An exploration zone was demarcated five body lengths (10 cm) from the refuge chamber. The maze itself consisted of two parallel barriers, requiring the fish to take two turns to traverse it, at the end of which a feeding ring was placed, into which food was dropped. The predator chamber was 10 cm in width and remained empty throughout the testing and training period except for the trial in which a predator was used. Individual fish were trained to emerge from the refuge and navigate the maze, using a food reward. While it is still not known how persistent long-term memory can be in zebrafish (Gerlai, 2016), earlier studies on the memory of spatially learned tasks show that adult wild zebrafish successfully performed spatial tasks up to 4 days after training (Roy and Bhat, 2018, 2018b). Taking this into account, subjects underwent eight training trials on consecutive days followed by a 3-day gap, after which they were tested for memory (trial 9). Following the memory test, performance was checked in the presence of a snakehead (*Channa* sps.), which is a predatory species commonly found with zebrafish, to further test for effects of a risk in completion of the task (trial 10). This final test (in the presence of a predator) was an additional means to test for consistency in individual performance under varying conditions, which would have an effect on repeatability. Each trial lasted for 15 min. The length of each trial, gap before testing for memory (3 days), as well as use of a predator to test change in behavior were chosen based on a pilot study that showed that this was a reasonable time gap for testing memory.

In each trial, fish were gently netted from the meshed isolation tank, placed into the refuge chamber in the experimental tank and allowed to acclimatize for 2 min, following which, the window in the chamber was opened for the fish to emerge into the experimental tank. The fish was allowed to swim through the window into the tank and food was dropped into the feeding ring placed at the end of maze once the fish crossed the exploration zone for all the trials. Food was dropped manually by the experimenter from the side of the tank to ensure minimal disturbance. Fish were returned into their individual mesh compartments at the end of the trial. Test fish were only fed in the experimental tank and not in the isolation tanks in order to maintain similar motivation levels throughout the experiment. Fish which failed to emerge or complete the feeding task in three consecutive trials were not considered for analysis. The predator was placed into the predator compartment of the experimental tank for a minimum of 12 h before the start of the predator trial to allow for homogenization of the chemical cues released by the predator in the tank and remained in a separate holding tank for the rest of the trials. All fish were tested in the same tank, and the same predator was used throughout the experiment to prevent any setup or predator related factors from contributing to the variation. Size of individuals was measured at the end of the experiment by placing each individual in a flat plastic bag containing water and photographing it with a ruler for scale. Total and standard body length was then obtained from the images using *ImageJ*. Each trial was recorded with a video camera placed vertically above the experimental tank (Sony DCR–SX22E or Sony DCR–PJ5E). All observations were done in a random sequence and by the same observer (DKD).

Final sample size for each population was obtained after eliminating individuals that did not respond to the opening of the window in the refuge chamber or died (two males, three females) during the course of the experiment, and was as follows–35 from CB (18 males, 17 females), 34 from KB (17 males, 17 females), and 32 from LK (16 males, 16 females).

### Measures Observed

The following variables were observed and tracked repeatedly across trials in order to obtain repeated measures of each behavioral trait:

• Time taken by the fish to emerge from the refuge chamber after the window was opened was taken as the **emergence latency** (measure of boldness in the first trial and indicative of habituation or familiarity to the setup in later trials).
• Time taken by fish to cross the exploration zone and move five body lengths away from the refuge chamber after emerging was taken as the **exploration latency** (measure of exploratory tendency in the first trial and indicative of habituation to the setup in later trials).
• Time taken by the fish to navigate the maze, reach the food ring and take a bite of the food after emergence from the chamber (referred to as the **feeding latency**). This provided a measure of spatial ability in the first trial and also of spatial task learning in later trials.

### Statistical Analysis

All statistical analyses were performed in R (ver. 4.1.0; R Core Team, 2021) run through RStudio (ver. 1.4.1106; RStudio Team, 2020). Data was fit to various distributions using *fitdistrplus* (Delignette-Muller and Dutang, 2015), and since it lay close to none of the known families (very high AIC values) no distribution was specifically considered for further analysis. We used the package *lmerTest* (Kuznetsova et al., 2017) to fit generalized linear mixed models (GLMMs) for all three behavioral measures, which were then used to calculate repeatability. Emergence latency, exploration latency and feeding latency were taken as the dependent variables and sex, size, trial and population were taken as the fixed factors, as were the interaction terms between these factors (sex*size, sex*trial, sex*population, size*trial, size*population, trial*population). Fish ID was taken as the random factor. These models were built for data pooled from all populations, as well as for the data for each population. For the latter, the same factors (except “population”) as those for the combined model were used. Significant factors and interactions were considered, and insignificant factors were removed, to obtain a simpler model. Models were compared using ANOVA performed using the *car* package, and if they were found to be parsimonious, then the simpler model was selected for further analysis.

Repeatability is the quantification of the proportion of variance caused by inter-individual differences (when individual ID is considered the random factor in mixed models) and is mathematically expressed as R = between-individual variance/(between-individual variance + residual variance) (Boake, 1989). To estimate repeatability across and within populations, the *rptR* package (Stoffel et al., 2017) was used, which calculates repeatability from random factors of generalized linear mixed models and estimates *p*-values and confidence intervals through Monte Carlo simulation methods.

Repeatability was first calculated without adjusting for the fixed factors in the model, by only including a random factor, which was Fish ID. The model therefore uses the simplest form of the aforementioned mathematical expression, only including between individual variance and residual variance in the denominator. Subsequently, fixed factors were also included in the models, in order to understand the proportion of variance caused due to them, and obtain the adjusted repeatability, which includes the variance caused by the fixed factors into the denominator. The *enhanced agreement repeatability* was also obtained, which includes variance explained by the fixed factors in the denominator but is different from the adjusted repeatability since it completely removes their contribution from the numerator (Stoffel et al., 2017). This is significant because some of the fixed factors (sex, size, population, and their interactions) themselves may contribute to the variance caused by interindividual differences. The coefficient of determination for fixed effects (*R*^2^) was also calculated, which allowed us to measure the proportion of variance contributed by the fixed factors. Since some of the significant factors in the model themselves may contribute to interindividual differences (size, sex, and population), they were also included as random factors, in order to determine how much of the repeatability was caused by them. We also compared repeatability within sexes both across and within populations. All estimates were obtained with 1,000 parametric bootstrap iterations and 1,000 randomizations for permutation-based null hypothesis testing (nboot = 1,000 and npermut = 1,000).

Once the repeatability values were obtained, we compared them by looking for overlap of confidence intervals. If there was no overlap, the repeatability estimates were considered different for that specific behavior. If repeatability was seen due to Fish ID, in order to understand which individual fixed factors (size, sex, population) caused consistent inter-individual variation, we then ran the same model with those as random factors along with Fish ID. The repeatability for these factors may explain how much of repeatability due to individual identity is caused by random variations between individuals, and how much depends on some other innate factor such as sex, size or population.

## Results

**FIGURE 2:**
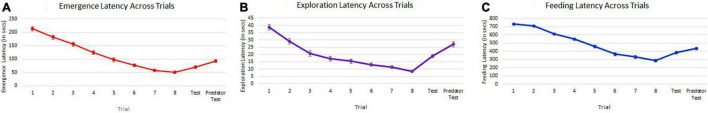
Decrease in emergence latency **(A)**, exploration latency **(B)**, and feeding latency **(C)** across the training trials (1–8) and then increase in test trials (9 and 10). Points on graphs represent means across populations and error bars represent the standard error.

GLMMs built for each of the behavioral measures indicate that all the three measures tested–emergence latency, exploration latency and feeding latency were significantly affected by population, size, sex (in interaction with other factors), and trial (Tables 1–3). The impact of trial on the behavioral measures also indicates that habituation (for personality related behaviors) and learning (for spatial task completion) has occurred, resulting in a reduction in latency for emergence, exploration as well as feeding (Table 4 and Figure 2). Interaction between size and population, sex and population, as well as population and trial were also significant for all behaviors. In addition, exploration latency was also affected by an interaction of size and trial, and feeding latency was affected by an interaction of sex and trial.

**TABLE 1:** Type III Analysis of variance table with Satterthwaite’s method for emergence latency across populations.

[Emergence Latency ∼ Size + Sex + Population + Trial + Size*Population + Sex*Population + Population*Trial + (1| Fish ID)]			
	***F*-value**	**Pr(>*F*)**	
Size	7.8245	0.006277	**
Sex	0.6916	0.407708	
Pop	13.0737	1.01E-05	***
Trial	537.4604	<2.2e-16	***
Size:pop	4.959	0.009009	**
Size:trial	5.0544	0.008236	**
Sex:pop	8.3736	4.35E-13	***
Pop:trial	55.1781	<2.2e-16	***

Significance codes: “***”0.001 “**”0.01.

**TABLE 2:** Type III analysis of variance table with Satterthwaite’s method for exploration latency across populations.

[Exploration Latency ∼ Size + Sex + Population + Trial + Size*Population + Size*Trial + Sex*Population + Population*Trial + (1| Fish ID)]			
	***F*-value**	**Pr(>*F*)**	
Size	21.566	1.12E-05	***
Sex	2.1585	0.145194	
Pop	25.5346	1.47E-09	***
Trial	15.8467	<2.2e-16	***
Size:pop	13.2996	8.37E-06	***
Size:trial	5.9025	1.13E-08	***
Sex:pop	4.9044	0.009465	**
Pop:trial	91.4269	<2.2e-16	***

Significance codes: “***”0.001 “**”0.01.

**TABLE 3:** Type III analysis of variance table with Satterthwaite’s method for feeding latency across populations.

[Feeding Latency ∼ Size + Sex + Population + Trial + Size*Population + Sex*Population + Sex*Trial + Population*Trial + (1| Fish ID)]			
	***F*-value**	**Pr(>*F*)**	
Size	29.2884	4.93E-07	***
Sex	1.5887	0.210631	
Pop	30.3691	7.45E-11	***
Trial	2087.016	<2.2e-16	***
Size:pop	7.53	0.000936	***
Sex:pop	10.0422	0.000113	***
Sex:trial	9.6163	2.54E-15	***
Pop:trial	226.8773	<2.2e-16	***

Significance codes: “***”0.001.

**TABLE 4:** Results from pairwise comparisons (Wilcoxon Rank sum test with continuity corrections) of behavior between trial 2 and last trial, indicating habituation and learning.

	Emergence latency	Exploration latency	Feeding latency
All populations	9246.5***	8984***	10194***
CB	1225***	1204***	1225***
LK	1024***	1021***	1024***
KB	1156***	1156***	1156***

Test Statistic is W, as obtained from paired Wilcoxon tests. (All populations: n = 101; CB: n = 35; KB: n = 34; LK: n = 32). Significance codes: “***”0.001.

**FIGURE 3:**
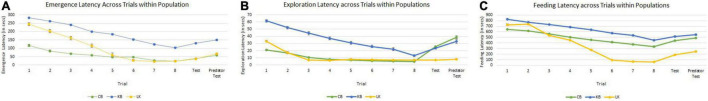
Comparison of decrease in emergence latency **(A)**, exploration latency **(B)**, and feeding latency **(C)** across the training trials (1–8) and then increase in test trials (9 and 10) for Cooch Behar (CB), Kali Bazar (KB), and Leturakhal (LK). Points on graphs represent means across populations and error bars represent the standard error.

Results of the GLMM built separately for each population revealed in the three populations, emergence latency depended on sex (individually in CB, and in interaction with trial in LK, but not in KB) and trial (Supplementary Tables 1.1–3.3 and Figure 3). In CB and KB, emergence latency also depended on size of the individuals. Exploration latency also significantly depended on size, sex and trial in CB; on size and trial in KB; and on sex and trial in LK. Feeding latency also showed trends similar to exploration latency and depended significantly on size, sex and trial in CB; on size and trial in KB; and on sex and trial in LK. Significant effect of trial in the models for all three behaviors in each of the three populations reiterates that habituation or learning is occurring (Table 4). However, the individual factors which determine behavior (such as sex and size) are different for the three populations as well as for each behavior within the population.

### Repeatability in Behaviors Across Populations

#### Emergence Latency

Emergence latency showed moderate repeatability without adjustment for fixed factors (Table 5) which did not change when it was adjusted with size, sex, population, trial, and their interaction as fixed factors. However, the enhanced agreement repeatability was very low for Fish ID, and most of the variance in the data seemed to be contributed by the fixed factors (Table 6).

**TABLE 5:** Repeatability for behaviors across populations with Fish ID as random factor.

Behavior	Fixed factors	Type of repeatability	R	SE	CI	*p*
Emergence latency	None	Non-adjusted	0.41	0.042	(0.323, 0.487)	<0.001
	Size, sex, population, trial	Adjusted	0.352	0.041	(0.279, 0.44)	<0.001
	Size, sex, population, trial	Enhanced agreement	0.041	0.007	(0.03, 0.058)	<0.001
Exploration latency	None	Non-adjusted	0.466	0.043	(0.376, 0.543)	<0.001
	Size, sex, population, trial	Adjusted	0.586	0.04	(0.517, 0.672)	<0.001
	Size, sex, population, trial	Enhanced agreement	0.092	0.014	(0.071, 0.123)	<0.001
Feeding latency	None	Non-adjusted	0.287	0.037	(0.212, 0.355)	<0.001
	Size, sex, population, trial	Adjusted	0.441	0.042	(0.37, 0.534)	<0.001
	Size, sex, population, trial	Enhanced agreement	0.02	0.003	(0.015, 0.029)	<0.001

*Standard errors (SE), 95% confidence intervals (CI) and p-values are provided.*

**TABLE 6:** Proportion of variance caused due to fixed factors during calculation of enhanced agreement repeatability for Fish ID across populations.

Behavior	Proportion of variance due to fixed factors	SE	CI
Emergence latency	0.884	0.008	(0.867, 0.897)
Exploration latency	0.843	0.014	(0.81, 0.865)
Feeding latency	0.954	0.004	(0.945, 0.959)

*Standard errors (SE) and 95% confidence intervals (CI) are provided.*

When size, sex, and population were included as random factors along with Fish ID to obtain the enhanced agreement repeatability, only population significantly explained the variance in data along with trial, which was a fixed factor (Table 7). Proportion of variance explained by Fish ID was also very low indicating that sex and size do not contribute to the interindividual differences, whereas population does.

**TABLE 7:** Proportion of variance contributed due to individual factors (random factors) as well as experimental conditions (fixed factors) for each behavior across populations.

Behavior	Factor	Type of factor	Proportion of variance explained	SE	CI	*p*
Emergence latency	Fish ID	Random	0.042	0.019	(0.016, 0.087)	<0.001
	Sex	Random	0.002	0.007	(0, 0.022)	0.157
	Size	Random	0	0.007	(0, 0.027)	1
	Population	Random	0.491	0.22	(0.015, 0.775)	<0.001
	Trial	Fixed	0.332	0.144	(0.144, 0.646)	NA
Exploration latency	Fish ID	Random	0.111	0.048	(0.049, 0.222)	<0.001
	Sex	Random	0.009	0.018	(0, 0.062)	0.089
	Size	Random	0	0	(0, 0)	0.5
	Population	Random	0.459	0.211	(0.022, 0.75)	<0.001
	Trial	Fixed	0.215	0.085	(0.1, 0.398)	NA
Feeding latency	Fish ID	Random	0.025	0.01	(0.008, 0.047)	<0.001
	Sex	Random	0.003	0.006	(0, 0.021)	0.058
	Size	Random	0	0.004	(0, 0.015)	0.5
	Population	Random	0.377	0.196	(0.012, 0.699)	<0.001
	Trial	Fixed	0.47	0.148	(0.225, 0.748)	NA

*Standard errors (SE), 95% confidence intervals (CI), and p-values are provided.*

Taking only population as a random factor along with Fish ID, and sex and size as fixed factors along with trial further confirmed the contribution of population to interindividual differences, as the enhanced agreement repeatability was similar for population (*R* = 0.428, *p* < 0.001) and fixed factors (*R* = 0.389), and least for Fish ID (*R* = 0.043, *p* < 0.001).

#### Exploration Latency

The repeatability for exploration time was moderate without adjustment (Table 5) and did not change when adjusted with size, sex, population, and trial as fixed factors. The enhanced agreement repeatability was extremely low, and the proportion of variance explained by fixed factors was quite high (Table 6).

When including size, sex, and population as random factors along with Fish ID, the enhanced agreement repeatability indicates that population shows significant repeatability, but not size and sex (Table 7). Fish ID and trial show similar repeatability to each other yet have less contribution to the overall variance.

When only population was taken as a random factor with Fish ID, it showed significant repeatability (*R* = 0.451, *p* < 0.001) which was comparable to the fixed factors (size, sex and trial; *R* = 0.321), although it was much higher than that for Fish ID (*R* = 0.091, *p* < 0.001).

#### Feeding Latency

Feeding latency was repeatable without adjustment (Table 5), but less than other traits. However, repeatability increased on adjustment by including size, sex, population and trial as fixed factors. The enhanced agreement repeatability was very low, and fixed factors explained a large proportion of variance in the data (Table 6).

For feeding latency, when individual characteristics such as sex, size and population are included as random factors with Fish ID, trial (as the fixed factor) has maximum contribution to the variance (Table 7). Among the random factors, only population shows repeatability, with Fish ID explaining a very low proportion of the variance.

When considering population and Fish ID as random factors and size, sex and trial as fixed factors, fixed factors (*R* = 0.458) and population (*R* = 0.412, *p* < 0.001) explain almost all the variation in feeding latency, with a very small contribution made by Fish ID (*R* = 0.015, *p* < 0.001).

### Comparison of Repeatability and Drivers of Interindividual Variation Within Populations

#### Emergence Latency

Repeatability in emergence time was only seen in CB without adjustment (Table 8). However, on including sex, size, and trial as fixed factors, all three populations showed significantly high repeatability (Table 8). Repeatability in KB and CB were similar to each other, and LK showed lower repeatability. Estimation of the enhanced agreement repeatability revealed that fixed factors (sex, size and trial for CB and KB; sex and trial for LK) contributed to most of the variance seen in emergence time for all three populations (Table 9). Repeatability due to Fish ID was extremely low in KB and LK and only slightly higher in CB (Table 8).

**TABLE 8:** Repeatability for behaviors within populations with Fish ID as random factor.

Behavior	Pop	Fixed factors	Type of repeatability	R	SE	CI	*p*
Emergence latency	CB	None	Non-adjusted	0.212	0.057	(0.095, 0.322)	<0.001
		Size, sex, trial	Adjusted	0.57	0.067	(0.446, 0.701)	<0.001
		Size, sex, trial	Enhanced agreement	0.11	0.029	(0.068, 0.179)	<0.001
	LK	None	Non-adjusted	0	0.016	(0, 0.052)	1
		Size, sex, trial	Adjusted	0.308	0.068	(0.192, 0.46)	<0.001
		Sex, trial	Enhanced agreement	0.067	0.021	(0.033, 0.113)	<0.001
	KB	None	Non-adjusted	0	0.017	(0, 0.06)	1
		Size, sex, trial	Adjusted	0.686	0.061	(0.543, 0.787)	<0.001
		Size, sex, trial	Enhanced agreement	0.059	0.015	(0.034, 0.094)	<0.001
Exploration latency	CB	None	Non-adjusted	0.039	0.03	(0, 0.105)	0.098
		Size, sex, trial	Adjusted	0.449	0.074	(0.314, 0.603)	<0.001
		Size, sex, trial	Enhanced agreement	0.059	0.017	(0.035, 0.1)	<0.001
	LK	None	Non-adjusted	0.016	0.023	(0, 0.075)	0.322
		Size, sex, trial	Adjusted	0.295	0.071	(0.167, 0.444)	<0.001
		Sex, trial	Enhanced agreement	0.069	0.021	(0.035, 0.118)	<0.001
	KB	None	Non-adjusted	0.267	0.064	(0.136, 0.393)	<0.001
		Size, sex, trial	Adjusted	0.705	0.058	(0.581, 0.808)	<0.001
		Size, sex, trial	Enhanced agreement	0.211	0.045	(0.136, 0.314)	<0.001
Feeding latency	CB	None	Non-ADJUSTED	0.102	0.045	(0.023, 0.201)	<0.001
		Size, sex, trial	Adjusted	0.449	0.074	(0.314, 0.603)	<0.001
		Size, sex, trial	Enhanced Agreement	0.08	0.02	(0.051, 0.128)	<0.001
	LK	None	Non-Adjusted	0	0.017	(0, 0.06)	1
		Size, sex, trial	Adjusted	0.124	0.051	(0.039, 0.237)	<0.001
		Size, sex, trial	Enhanced Agreement	0.004	0.002	(0.001, 0.009)	<0.001
	KB	None	Non-Adjusted	0.062	0.036	(0, 0.14)	0.02
		Size, sex, trial	Adjusted	0.78	0.045	(0.69, 0.864)	<0.001
		Size, sex, trial	Enhanced Agreement	0.059	0.015	(0.036, 0.091)	< 0.001

*Standard errors (SE), 95% confidence intervals (CI), and p-values are provided.*

**TABLE 9:** Proportion of variance caused due to fixed factors during calculation of enhanced agreement repeatability for Fish ID within populations.

Behavior	Population	Proportion of variance due to fixed factors	SE	CI
Emergence latency	CB	0.807	0.028	(0.741, 0.852)
	LK	0.783	0.022	(0.737, 0.825)
	KB	0.914	0.015	(0.88, 0.939)
Exploration latency	CB	0.869	0.016	(0.83, 0.894)
	LK	0.766	0.021	(0.721, 0.806)
	KB	0.701	0.044	(0.608, 0.779)
Feeding latency	CB	0.888	0.02	(0.842, 0.919)
	LK	0.967	0.003	(0.96, 0.972)
	KB	0.914	0.015	(0.881, 0.939)

*Standard errors (SE) and 95% confidence intervals (CI) are provided.*

Within populations also, significant traits included in fixed factors were not the same (size and sex for CB and KB; only sex for LK), due to which we explored the variance contributed by them. In CB, size was not significantly repeatable (*R* = 0.118, *p* = 0.208), but sex was (*R* = 0.256, *p* < 0.001), with the repeatability in Fish ID remaining very low (*R* = 0.011, *p* < 0.001), indicating that a large proportion of the individual variance is caused by sex differences. Since repeatability was very low for KB and LK, we did not check for effect of size and sex.

#### Exploration Latency

Without adjusting for fixed factors, the repeatability for CB and LK were comparably low and insignificant, but significantly higher in KB (Table 9). On adjustment with size, sex and trial, enhanced agreement repeatability due to Fish ID remained low, but was significant for CB and LK, and remained the same for KB (Table 8). The effect of fixed factors was maximum in CB and comparably high in KB and LK (Table 9).

On delving into the effect of traits contributing to individual variations (size and sex in CB; only size in KB; only sex in LK), we found although size did not have significant contribution to repeatability (*R* = 0.058, *p* = 0.416), sex did have a slight contribution (*R* = 0.117, *p* < 0.001). However, on removing the effect of sex, Fish ID no longer showed significant repeatability, implying that all interindividual variation in exploration time is caused by sex in CB. In KB, size had no effect [*R* = 0.099, CI = (0, 0.354), *p* = 0.281] and Fish ID remained significantly repeatable (*R* = 0.231, *p* < 0.001), but in LK, sex (*R* = 0.052, *p* = 0.006) and Fish ID (*R* = 0.067, *p* < 0.001) both were significant, indicating random interindividual variation due to other unknown factors in both populations.

#### Feeding Latency

Without adjustment, feeding latency shows repeatability across Fish ID in CB but not in KB and LK (Table 8). Upon adjusting with size, sex and trial and calculating the enhanced agreement repeatability, there was very low repeatability across Fish ID for CB, LK and KB (Table 8). Fixed factors had a very high contribution in all three populations with LK showing the highest proportion of variance caused by fixed factors, and that of CB and KB being comparable (Table 9). Since there was no repeatability across Fish ID in any of the populations, we did not test for other factors which might contribute to it.

## Discussion

The major aim of our study was to investigate inter- and intra-individual variations in specific behaviors when they are subjected to the effects of learning and habituation within and across three wild zebrafish populations. These populations belonged to habitats which differed in flow regime and turbidity (CB had highest flow and lowest turbidity, LK had moderate flow and turbidity and KB had least flow and highest turbidity). Furthermore, if certain behaviors are repeatable within individuals, we enquired whether this repeatability was consistent across populations and whether it depended on population or individual factors (such as size or sex). Our results revealed, that even in behaviors involving habituation (such as emergence or exploration) or learning (performance in a spatial task), there are inter-individual differences in performance, and these differences persist over time both within and across populations. However, for a behavior like feeding latency in a maze, where performance in the task significantly improves over time, these inter-individual differences are less important contributors to the variance in the data compared to the effect of trial. In all behaviors, trial was the most significant fixed factor, indicating that change in behavior over time had a more significant effect on behavior than individual consistency. Native population contributed significantly to the overall repeatability, and within population repeatability, although lower, was governed by different individual factors (size and sex) for each population.

Patterns in behavioral variation considering pooled observations from all three populations, were found to be different from the patterns within each population. Although the three populations are in habitats that vary across a gradient of flow and water turbidity regimes, there are also other differences between them (such as habitat complexity due to substratum and vegetation, food availability, predation pressure, etc.) which might contribute to the differences in behavior (Fraser et al., 2001; Laskowski et al., 2015). The extent of variation between individuals and consistency within individuals (represented by repeatability) in each population differs from other populations. This, in turn, could significantly contribute to the overall inter-individual variation seen in behavior, since population was the most significant individual trait that affected repeatability.

Within populations, behaviors differed with respect to the extent of variations and the factors that contributed to these variations. There was highest overlap between CB and KB (emergence, exploration, and feeding latency), indicating similar selection pressures operating on these populations. Although the two habitats are vastly different from each other, they are both immensely dynamic in terms of seasonal variation, as opposed to LK which remains stable throughout the year. The CB habitat shows changes in water volume, flow strength and routinely shifts its course. On the other hand, KB habitat is an ephemeral (seasonal) stream, and appears during the monsoon, but dries up over the year and nearly disappears during the summer. As the latter habitat is replenished from multiple sources (runoff from agricultural fields, natural streams, and manmade channels), it also shows differences in water turbidity and chemical properties across years, depending on from where it receives water during the monsoon.

In nature, behavior of wild populations is strongly driven by changes to habitat conditions. A study on southern black racers (*Coluber constrictor priapus*) showed that habitat disturbances caused by prescribed fires significantly changed their behavior and distribution of the snakes, with greater surface activity and bolder personalities in burnt areas (Howey et al., 2016). Black capped chickadees (*Poecile atricapillus*) inhabiting fragmented forests showed greater resilience to climate change, as they had higher levels of metabolic sensitivity (Latimer et al., 2018). Even ecosystem studies in pelagic ecosystems show that habitat variability significantly changes the productivity and resource use efficiency of plankton (Kemp et al., 2001). Nightjars (*Caprimulgus europaeus*) facing greater habitat change show more variability in behavior over time (Mitchell et al., 2020). Although habitat features themselves might influence behavior and repeatability of behavior, it might be that the variability of the habitat has a much higher impact, which would explain why more dynamic habitats are similar to each other in our study, at least in terms of repeatability.

Our results also revealed differences in the underlying factors contributing to inter-individual variation within populations. Sex differences were found to play a significant role toward both emergence latency and exploration latency, and thus, were possibly the cause for much of the inter-individual differences observed in our study. As sexual selection is believed to exert different pressures on males and females, this can result in sex-based differences in specific animal personality traits (i.e., differences in the way individuals vary in behavior) that are under selection (Schuett et al., 2010). Studies on chimpanzees, capuchins, and zebra finches have shown that there are sex differences in personality (Buirski et al., 1978; Schuett and Dall, 2009; Manson and Perry, 2013). These differences would increase the overall variability in behavior across the species, since males and females are inherently different from each other, as was also shown in a previous study (Daniel and Bhat, 2020). However, studies have also indicated that not all species or populations differ in personality across sexes (Budaev, 1999; Harrison et al., 2021). The random variations between individuals in their exploration latencies for LK populations, not explained by size or sex, could be due to underlying genetic, physiological, or even neurological differences that we have not considered (Carlson and Glick, 1989; Llera et al., 2019).

Therefore, our study shows that even as repeatability in behavior is present in tasks that show changes over time due to habituation, the extent is lower in tasks where improvement in performance due to learning occurs. Under the influence of training or acclimatization to the test setup, the behavior in question is far more dependent on the length or rigor of the training rather than any inherent inter-individual difference in behavior. However, when individual differences do significantly contribute to variation, they are driven by population level differences. We also found that within populations, variations are mostly driven by sex differences. Although further analysis is required in order to quantify intra-individual variation and to control for the changes in behavior wrought by training, this study elucidates some of the proximate causes for inter-individual variation in behavior in both personality and cognition related tasks. Extended studies that also include more population replicates for each kind of habitat could provide further understanding of the evolutionary underpinnings of the role of habitat dependent characteristics in shaping behavioral variations at intra- and inter-population levels.

## Data Availability Statement

The raw data supporting the conclusions of this article will be made available by the authors, without undue reservation.

## Ethics Statement

The animal study was reviewed and approved by Institutional Animal Ethics Committee’s (IAEC) approval (Approval number IISERK/IAEC/AP/2021/70), Indian Institute of Science Education and Research Kolkata, India.

## Author Contributions

DD conducted the experimental assays, analyzed the videos, performed the statistical analyses, and wrote the first draft of the manuscript. AB procured funding and supervised the project. Both authors conceived the goals and the design of the study, wrote and edited the final version of manuscript, and read and approved the submitted version.

## Conflict of Interest

The authors declare that the research was conducted in the absence of any commercial or financial relationships that could be construed as a potential conflict of interest.

## Publisher’s Note

All claims expressed in this article are solely those of the authors and do not necessarily represent those of their affiliated organizations, or those of the publisher, the editors and the reviewers. Any product that may be evaluated in this article, or claim that may be made by its manufacturer, is not guaranteed or endorsed by the publisher.
