# Supplementary material for: Sex and Population Drive Interindividual Variations in a Cognitive Task Across Three Populations of Wild Zebrafish
Source: Front Psychol. 2022 Mar 4;13:786486. doi: 10.3389/fpsyg.2022.786486 (PMC8931718; doi:10.3389/fpsyg.2022.786486)
Supplement: Supplementary file 1 [file Table_1.DOCX]

Supplementary Table 1.1. Type III Analysis of Variance Table with Satterthwaite's method for Emergence Latency in CB

(Emergence Latency ~ Size + Sex + Trial + Size*Trial + Sex*Trial + (1|Fish ID)

|  | **F value** | **Pr(>F)** |  |
| --- | --- | --- | --- |
| **size** | 12.3647 | 0.001332 | ** |
| **sex** | 8.8207 | 0.005608 | ** |
| **trial** | 12.2898 | < 2.2e-16 | *** |
| **size:trial** | 5.2478 | 1.27E-06 | *** |
| **sex:trial** | 6.4157 | 2.62E-08 | *** |

*Significance codes: ‘***’0.001 ‘**’0.01 ‘*’ 0.05 ‘.’0.1 ‘ ’ 1*

Supplementary Table 1.2. Type III Analysis of Variance Table with Satterthwaite's method for Exploration Latency in CB

(Exploration Latency ~ Size + Sex + Trial + Size*Trial + Sex*Trial + (1|Fish ID)

|  | **F value** | **Pr(>F)** |  |
| --- | --- | --- | --- |
| **size** | 9.2139 | 0.004744 | ** |
| **sex** | 6.1697 | 0.018421 | * |
| **trial** | 12.7034 | < 2.2e-16 | *** |
| **size:trial** | 4.1497 | 4.79E-05 | *** |
| **sex:trial** | 2.0661 | 0.032527 | * |

*Significance codes: ‘***’0.001 ‘**’0.01 ‘*’ 0.05 ‘.’0.1 ‘ ’ 1*

Supplementary Table 1.3. Type III Analysis of Variance Table with Satterthwaite's method for Feeding Latency in CB

(Feeding Latency ~ Size + Sex + Trial + Size*Trial + (1|Fish ID)

|  | **F value** | **Pr(>F)** |  |
| --- | --- | --- | --- |
| **size** | 3.2711 | 0.000944 | *** |
| **sex** | 6.3429 | 0.016981 | * |
| **trial** | 8.4916 | 2.63E-11 | *** |
| **size:trial** | 9.5393 | 8.82E-13 | *** |

*Significance codes: ‘***’0.001 ‘**’0.01 ‘*’ 0.05 ‘.’0.1 ‘ ’ 1*

Supplementary Table 2.1. Type III Analysis of Variance Table with Satterthwaite's method for Emergence Latency in KB

(Emergence Time ~ Size + Sex + Trial + (1|Fish ID)

|  | **F value** | **Pr(>F)** |  |
| --- | --- | --- | --- |
| **size** | 17.463 | 0.000222 | *** |
| **sex** | 3.1783 | 0.084419 | . |
| **trial** | 1221.594 | < 2.2e-16 | *** |

*Significance codes: ‘***’0.001 ‘**’0.01 ‘*’ 0.05 ‘.’0.1 ‘ ’ 1*

Supplementary Table 2.2. Type III Analysis of Variance Table with Satterthwaite's method for Exploration Latency in KB

(Exploration Time ~ Size + Trial + Size*Trial + (1|Fish ID)

|  | **F value** | **Pr(>F)** |  |
| --- | --- | --- | --- |
| **size** | 18.5712 | 0.000146 | *** |
| **trial** | 6.6503 | 1.2E-08 | *** |
| **size:trial** | 3.0752 | 0.001542 | ** |

*Significance codes: ‘***’0.001 ‘**’0.01 ‘*’ 0.05 ‘.’0.1 ‘ ’ 1*

Supplementary Table 2.3. Type III Analysis of Variance Table with Satterthwaite's method for Feeding Latency in KB

(Feeding Time ~ Size + Sex + Trial + Size*Trial + Sex*Trial + (1|Fish ID)

|  | **F value** | **Pr(>F)** |  |
| --- | --- | --- | --- |
| **size** | 21.1155 | 6.83E-05 | *** |
| **trial** | 7.7009 | 4.1E-10 | *** |
| **sex** | 2.9592 | 0.095359 | . |
| **size:trial** | 2.5152 | 0.008768 | ** |
| **trial:sex** | 3.1197 | 0.001354 | ** |

*Significance codes: ‘***’0.001 ‘**’0.01 ‘*’ 0.05 ‘.’0.1 ‘ ’ 1*

Supplementary Table 3.1. Type III Analysis of Variance Table with Satterthwaite's method for Emergence Latency in LK

(Emergence Latency ~ Sex + Trial + Sex*Trial + (1|Fish ID)

|  | **F value** | **Pr(>F)** |  |
| --- | --- | --- | --- |
| sex | 2.6826 | 0.1119 |  |
| trial | 174.0208 | < 2.2e-16 | *** |
| sex:trial | 8.9859 | 7.27E-12 | *** |

*Significance codes: ‘***’0.001 ‘**’0.01 ‘*’ 0.05 ‘.’0.1 ‘ ’ 1*

Supplementary Table 3.2. Type III Analysis of Variance Table with Satterthwaite's method for Exploration Latency in LK

(Exploration Latency ~ Sex + Trial + Sex*Trial + (1|Fish ID)

|  | **F value** | **Pr(>F)** |  |
| --- | --- | --- | --- |
| sex | 10.9033 | 0.002486 | ** |
| trial | 155.3972 | < 2.2e-16 | *** |
| sex:trial | 2.6306 | 0.006229 | ** |

*Significance codes: ‘***’0.001 ‘**’0.01 ‘*’ 0.05 ‘.’0.1 ‘ ’ 1*

Supplementary Table 3.3. Type III Analysis of Variance Table with Satterthwaite's method for Feeding Latency in LK

(Feeding Latency ~ Sex + Trial + Sex*Trial + (1|Fish ID)

|  | **F value** | **Pr(>F)** |  |
| --- | --- | --- | --- |
| **sex** | 20.093 | 1E-04 | *** |
| **trial** | 1148.861 | < 2.2e-16 | *** |
| **sex:trial** | 17.612 | < 2.2e-16 | *** |

*Significance codes: ‘***’0.001 ‘**’0.01 ‘*’ 0.05 ‘.’0.1 ‘ ’ 1*
